# Secure and Transparent Lung and Colon Cancer Classification Using Blockchain and Microsoft Azure

**DOI:** 10.3390/arm92050037

**Published:** 2024-10-17

**Authors:** Entesar Hamed I. Eliwa, Amr Mohamed El Koshiry, Tarek Abd El-Hafeez, Ahmed Omar

**Affiliations:** 1 Department of Mathematics and Statistics, College of Science, King Faisal University, P.O. Box 400, Al-Ahsa 31982, Saudi Arabia; 2 Department of Computer Science, Faculty of Science, Minia University, El Minia 61519, Egypt; 3 Department of Curricula and Teaching Methods, College of Education, King Faisal University, P.O. Box 400, Al-Ahsa 31982, Saudi Arabia; aalkoshiry@kfu.edu.sa; 4 Faculty of Specific Education, Minia University, El-Minia 61519, Egypt; 5 Computer Science Unit, Deraya University, El-Minia 61765, Egypt

**Keywords:** lung cancer, colon cancer, blockchain technology, Microsoft Azure, cloud services, convolutional neural networks (CNN), real-time diagnosis, secure remote consultations

## Abstract

**Highlights:**

The study presents a novel framework for remote consultation and lung and colon cancer classification, leveraging blockchain technology and Microsoft Azure cloud services to ensure data privacy and security. The proposed framework achieves an impressive accuracy of 100% for lung and colon cancer classification using advanced machine learning models, demonstrating its potential to improve diagnostic accuracy and streamline cancer care.

**What are the main findings?**

- **Effective Cancer Classification:** The framework effectively classifies lung and colon cancer using state-of-the-art machine learning models, achieving high accuracy, precision, recall, and *F*1-*score*.
- **Enhanced Data Security:** Blockchain technology and Microsoft Azure cloud services provide a secure and transparent environment for data storage, access, and sharing, ensuring patient privacy and data integrity.

**What is the implication of the main finding?**

- **Improved Diagnostic Efficiency:** The proposed framework has the potential to significantly improve the efficiency of lung and colon cancer diagnosis by enabling remote consultations and providing accurate and timely results.
- **Enhanced Patient Outcomes:** By improving diagnostic accuracy and streamlining the cancer care process, this framework can contribute to better patient outcomes and reduce the overall burden of lung and colon cancers.

**Abstract:**

Background: The global healthcare system faces challenges in diagnosing and managing lung and colon cancers, which are significant health burdens. Traditional diagnostic methods are inefficient and prone to errors, while data privacy and security concerns persist. Objective: This study aims to develop a secure and transparent framework for remote consultation and classification of lung and colon cancer, leveraging blockchain technology and Microsoft Azure cloud services. Dataset and Features: The framework utilizes the LC25000 dataset, containing 25,000 histopathological images, for training and evaluating advanced machine learning models. Key features include secure data upload, anonymization, encryption, and controlled access via blockchain and Azure services. Methods: The proposed framework integrates Microsoft Azure’s cloud services with a permissioned blockchain network. Patients upload CT scans through a mobile app, which are then preprocessed, anonymized, and stored securely in Azure Blob Storage. Blockchain smart contracts manage data access, ensuring only authorized specialists can retrieve and analyze the scans. Azure Machine Learning is used to train and deploy state-of-the-art machine learning models for cancer classification. Evaluation Metrics: The framework’s performance is evaluated using metrics such as accuracy, precision, recall, and *F*1-*score*, demonstrating the effectiveness of the integrated approach in enhancing diagnostic accuracy and data security. Results: The proposed framework achieves an impressive accuracy of 100% for lung and colon cancer classification using DenseNet, ResNet50, and MobileNet models with different split ratios (70–30, 80–20, 90–10). The *F*1-*score* and k-fold cross-validation accuracy (5-fold and 10-fold) also demonstrate exceptional performance, with values exceeding 99.9%. Real-time notifications and secure remote consultations enhance the efficiency and transparency of the diagnostic process, contributing to better patient outcomes and streamlined cancer care management.

## 1. Introduction

As healthcare increasingly moves towards digital solutions, the need for advanced, secure, and efficient systems for diagnosing and managing diseases has never been more critical [1,2,3,4]. Lung and colon cancers, which represent significant health burdens worldwide, require both accurate and timely diagnosis to improve patient outcomes and survival rates [5,6,7]. Traditional diagnostic methods for these cancers, which often involve manual analysis of CT scans and pathology slides, face several challenges, including inefficiencies, high error rates, and issues related to data privacy and security. Addressing these challenges is crucial for advancing cancer care and ensuring that patients receive the best possible treatment [8,9,10].

In recent years, the integration of cloud computing and blockchain technology has shown great promise in various sectors, including healthcare. Microsoft Azure, a leading cloud computing platform, offers a range of services designed to handle, store, and analyze large volumes of data securely. Its scalability and flexibility make it an ideal choice for managing medical imaging data and running complex machine-learning models. Blockchain technology [11], known for its decentralized and immutable ledger, provides a robust solution for ensuring data integrity, security, and transparency. Combining these technologies can address the critical issues of data privacy, security, and diagnostic accuracy in cancer care.

This research introduces a novel framework that integrates Microsoft Azure’s cloud services with blockchain technology to enhance the classification of lung and colon cancers. The framework leverages the decentralized nature of blockchain to ensure the privacy and transparency of sensitive patient data. By using blockchain, all data transactions, including the storage, sharing, and analysis of medical images, are recorded in an immutable ledger. This guarantees that the cancer diagnostic data cannot be tampered with, ensuring transparency in how data are handled. Additionally, blockchain’s cryptographic protocols provide secure, permissioned access to authorized users, safeguarding patient privacy during remote consultations and the classification process. This combination enhances the accuracy of cancer detection while maintaining the confidentiality and integrity of patient information.

Blockchain’s cryptographic protocols provide secure, permissioned access to authorized users, safeguarding patient privacy during remote consultations and the classification process. Additionally, smart contracts enable automated, rule-based access control, allowing only authorized individuals to access sensitive medical data under specific conditions. Patient data are encrypted before storage, and only individuals with the appropriate private key can decrypt it, reducing the risk of breaches. Moreover, blockchain integrates with external authentication mechanisms, such as multi-factor authentication (MFA), to further protect medical records and diagnostic images, enhancing data security and access control [12].

The framework operates in several stages, starting with the secure upload of lung and colon CT scans through a mobile application. Patients can use their smartphones or tablets to upload their scans directly to Azure Blob Storage, a secure and scalable cloud storage solution. Upon upload, Azure Functions automatically preprocess the data, including validating file formats and correcting metadata errors. Azure Cognitive Services’ Content Moderator then anonymizes the CT scans using techniques such as tokenization, which replaces sensitive patient information with random identifiers. This step is crucial for maintaining patient confidentiality while ensuring the data remains usable for medical analysis.

Once anonymized, the CT scan data are encrypted and stored securely in Azure Blob Storage. Integration with a permissioned blockchain network adds a layer of security by providing a tamper-proof ledger for managing data access and sharing. Smart contracts on the blockchain network control who can access the data, ensuring that only authorized specialists can retrieve and analyze the scans. These smart contracts also log access attempts and data usage, providing an auditable trail that enhances transparency and accountability.

Specialists access the system through a secure web portal, where Azure Active Directory manages user authentication and authorization. Upon receiving patient consent, specialists can retrieve anonymized CT scan data from Azure Blob Storage based on the access controls established by the smart contracts. For in-depth analysis, specialists utilize Azure Synapse Analytics, a powerful tool for data exploration and visualization. Real-time notifications, managed by Azure Notification Hub, keep both specialists and patients informed throughout the consultation process, ensuring timely and effective communication.

For cancer classification [13], the framework leverages Azure Machine Learning to train and deploy advanced machine learning models. Patients upload their CT images via a user-friendly interface, and these images are processed and analyzed using pre-trained models such as InceptionV3, ResNet, DenseNet, and MobileNet. These models are trained on the LC25000 dataset, a comprehensive collection of histopathological images used for evaluating cancer classification algorithms. The models are deployed as web services using Azure Functions, which handle API calls and manage access to the classification system.

The classification results are then communicated back to the mobile app or web interface, providing patients with clear and actionable insights into their cancer status. This process emphasizes the importance of consulting qualified medical professionals for final diagnosis and treatment, ensuring patients receive appropriate care based on the classification results.

The proposed framework represents a significant advancement in cancer diagnosis by integrating cloud computing and blockchain technology. It addresses the critical issues of data privacy, security, and diagnostic accuracy, providing a comprehensive solution that enhances the efficiency and effectiveness of lung and colon cancer classification. By combining Microsoft Azure’s (version number: 2025-01-05) scalable cloud services with blockchain’s secure and transparent data management capabilities, this framework aims to improve patient outcomes and streamline the diagnostic process, ultimately contributing to more effective cancer care and management.

### 1.1. Problem Statement

Traditional methods for lung and colon cancer diagnosis can be time-consuming, prone to errors, and often involve multiple physical consultations, which can be a significant burden for patients. Moreover, the increasing volume of medical data and the sensitivity of patient information necessitate robust solutions for secure data storage, processing, and sharing among medical professionals. There is a critical need for a framework that can enable secure, efficient, and remote consultations for lung and colon cancer diagnosis while ensuring the confidentiality and integrity of patient data.

### 1.2. Research Question

How can blockchain technology and Microsoft Azure be leveraged to develop a secure and transparent framework for the remote consultation and classification of lung and colon cancer, ensuring data security, patient privacy, and diagnostic accuracy?

### 1.3. Research Gap

While there have been significant advancements in the application of cloud computing and machine learning for medical image analysis, the integration of blockchain technology for enhancing data security and transparency in this domain is relatively underexplored. Existing studies have primarily focused on either the technical aspects of machine learning models for image classification or the potential of blockchain for secure data management without fully addressing the combined potential of these technologies for improving lung and colon cancer diagnosis processes.

### 1.4. Contributions

The proposed framework makes several noteworthy contributions to the field of medical imaging and cancer diagnosis:

1. Secure Remote Consultations: By implementing a blockchain-based permissioned network and leveraging Azure cloud services, the framework ensures secure and efficient remote consultations for lung and colon CT scans, addressing the challenges of data security and patient privacy.
2. Data Anonymization and Privacy Preservation: Utilizing Azure Cognitive Services for data anonymization, the framework prioritizes patient privacy without compromising the usability of medical data for diagnostic purposes.
3. Transparent and Controlled Data Access: Through the use of smart contracts on the blockchain network, the framework establishes transparent and controlled access to anonymized patient data for authorized specialists, enhancing trust in the remote consultation process.
4. Advanced Cancer Classification Models: Leveraging the capabilities of Azure Machine Learning, the framework implements state-of-the-art machine learning models for accurate classification of lung and colon cancer, potentially improving diagnostic accuracy and patient outcomes.
5. Scalability and Flexibility: The cloud-based architecture offers scalability and flexibility, accommodating increasing volumes of medical data and enabling the integration of additional functionalities over time.

This comprehensive approach, combining blockchain and cloud computing with advanced machine learning models, presents a significant step forward in the development of secure, efficient, and transparent systems for lung and colon cancer diagnosis and consultation.

## 2. Related Work

In the following section, we present some recent studies that have employed advanced deep-learning models and optimization algorithms to improve cancer detection and classification across various medical image datasets.

Bhattacharya et al. [14] extracted deep features from LC25000 images using pre-trained Convolutional Neural Networks (CNNs), specifically ResNet-18 and EfficientNet-b4-wide. This was followed by the application of the AdBet-WOA algorithm, which combined the Whale Optimization Algorithm and Adaptive β-Hill Climbing for effective feature selection. The proposed AdBet-WOA algorithm achieved remarkable performance, with an accuracy of 99.99% for the two-class classification of colon cancer, 99.97% for the 3-class classification of lung cancer, and 99.96% for the five-class classification combining both lung and colon datasets. These results indicated a significant improvement over previous methods, showcasing the effectiveness of the hybrid optimization approach in enhancing classification accuracy while reducing feature redundancy.

Dabass et al. [15] employed a Hybrid U-Net model enhanced with advanced methodologies, including Advanced Convolutional Learning Modules (ACLMs) for improved feature learning, Attention Modules (AMs) for refining features in skip connections, and Multi-Scalar Transitional Modules (MTMs) to prevent resolution degradation. Trained using a multi-task learning approach, the model simultaneously performed gland segmentation and cancer grade classification, benefiting from preprocessing techniques like patch extraction, data augmentation, stain normalization, and data cleaning. Applied to the LC-25000 dataset, the model achieved impressive results, with a 0.913 *F*1-*score* and 99.98% accuracy in cancer grade prediction. The Hybrid U-Net demonstrated strong generalizability and robustness, making it a valuable tool for assisting pathologists in diagnosing colorectal cancer through accurate morphological assessments and cancer classifications.

Mengash et al. [7] utilized a dataset comprising images for lung and colon cancer classification, dividing it into training and testing sets with an 80:20 ratio. The study employed the Marine Predators Algorithm (MPA) for hyperparameter tuning, integrated with deep learning techniques to develop the MPADL-LC3 model. This model was trained and tested using images specifically related to lung and colon cancers, with the MPA enhancing its performance compared to existing deep learning methods. Performance was evaluated using metrics such as accuracy, precision, recall, and F-score, with the MPADL-LC3 model achieving an impressive accuracy of 99.27%, precision of 98.18%, recall of 98.17%, and an F-score of 98.17%. Comparative analysis showed that other models, such as mSRC, Faster R-CNN, and RESNET-50, had lower performance metrics. The study demonstrated the potential of marine predator-inspired algorithms combined with deep learning to improve cancer diagnosis accuracy.

Halder and Dey [16] utilized the LC25000 dataset for training and validating the proposed MorphAttnNet framework. The MorphAttnNet framework integrated morphological operations with deep learning techniques to classify lung cancer subtypes, employing an attention mechanism to enhance the performance of both convolutional and morphological operations, thereby allowing the model to focus on relevant features in the images. Mathematical morphology was incorporated to analyze the topological structures of the regions of interest (ROIs) in the histopathological images. The framework was designed to classify different lung cancer subtypes, including benign, adenocarcinoma, and squamous cell carcinoma, by leveraging the morphological characteristics of the cells. The MorphAttnNet framework achieved a classification accuracy of 98.04% on the LC25000 dataset, significantly outperforming existing models such as VGG-16 (83.64%), VGG-19 (87.20%), and ResNet-50 (90.72%). The effectiveness of MorphAttnNet in accurately classifying lung cancer subtypes was demonstrated through various performance metrics. The study highlighted the potential of combining morphological operations with deep learning and attention mechanisms to improve the accuracy of lung cancer subtype classification from histopathological images.

Mohammad et al. [17] utilized a Kaggle dataset of brain MRI scans designed for brain tumor classification. It developed a blockchain-based Convolutional Neural Network (CNN) model to enhance prediction accuracy and security. Blockchain technology was integrated to secure and maintain the integrity of the CNN model. Features were extracted from MRI scans using the CNN and optimized with Genetic Algorithms (GA) to create a fused feature map, improving prediction accuracy by filtering out irrelevant features. The model’s performance was evaluated with various classifiers, where the Linear Discriminant (LD) classifier provided the highest results. Robustness was tested against different attack types (mild, average, and severe), demonstrating that blockchain integration improved resilience. The model achieved a prediction accuracy of 99.75%, precision of 97.94%, and recall of 98.73% with the LD classifier. It consistently outperformed traditional models like Inception v3 and GoogleNet, which had accuracies of 83.45% and 84.27%, respectively. The study highlighted the potential of combining blockchain with deep learning to enhance brain tumor prediction accuracy and security.

Heidari et al. [18] utilized four distinct datasets for training and testing the lung cancer detection method: the Cancer Imaging Archive (CIA), Kaggle Data Science Bowl (KDSB), LUNA 16, and a local dataset, comprising a total of 15,414 images, with 7954 labeled as cancerous and 7460 as normal. The images, collected in DICOM format, included various imaging modalities such as CT scans. A binary classification model, FBCLC-Rad, was developed using deep learning techniques, specifically leveraging Capsule Networks (CapsNet) for improved detection rates. Federated Learning (FL) allowed the model to be trained collaboratively across multiple hospitals while maintaining data privacy, enabling learning from shared data sources without transferring sensitive patient information. Blockchain technology ensured data integrity and security during the training process, facilitating a trustworthy environment for data sharing among institutions. Transfer Learning (TL) techniques were applied to enhance the performance of the deep learning models, improving their generalization across different datasets. The FBCLC-Rad model achieved a high accuracy of 99.69% on the local dataset. The study concluded that combining Federated Learning and blockchain technology significantly enhanced the accuracy and reliability of lung cancer detection from CT images while also addressing privacy concerns associated with patient data sharing.

Alabdulqader et al. [19] employed various deep learning and transfer learning models, specifically EfficientNetB4, CNN, VGG16, ResNet, MobileNet, Xception, and Inception-ResNetV2. These models were trained using two feature sets: the complete feature set of the cancer image dataset and Local Binary Pattern (LBP) features, which effectively captured texture information in images. The results indicated that the EfficientNetB4 model achieved the highest accuracy of 99.98% when using LBP features, with precision, recall, and F1 scores all at 99.99%. A 5-fold cross-validation was employed, yielding an average accuracy of 99.88%. The use of Shapley additive explanations (SHAP) enhanced the interpretability of the model’s predictions, providing insights into the contributions of individual features to the classification outcomes. Overall, the study demonstrated the effectiveness of combining deep learning models with texture-based features for accurate cancer detection in histopathological images. Table 1 summarizes the previous related works:

Our study distinctly builds upon the existing literature by integrating cloud computing and blockchain technology in a unified framework aimed at enhancing the accuracy, security, and efficiency of lung and colon cancer classification. While previous works, such as those by Bhattacharya et al. [14] and Halder and Dey [16], primarily focused on improving classification accuracy using advanced deep learning models and optimization algorithms, our framework incorporates a novel approach that emphasizes data privacy and secure remote consultations. Unlike studies that primarily utilize machine learning techniques without addressing the critical issues of data handling and patient privacy, our approach leverages blockchain’s immutable ledger and smart contract capabilities to ensure secure, permissioned access to sensitive medical data. Additionally, we introduce the use of Azure’s cloud services not only for data storage and processing but also for facilitating automated workflows, such as data preprocessing and real-time notifications, which enhances the user experience for both patients and specialists. By addressing these critical aspects, our research not only contributes to the ongoing advancements in cancer diagnosis but also sets a new standard for integrating security and privacy in healthcare solutions.

## 3. The Proposed Framework

### 3.1. Secure Remote Consultations of Lung and Colon CT Scans

**Figure 1:**
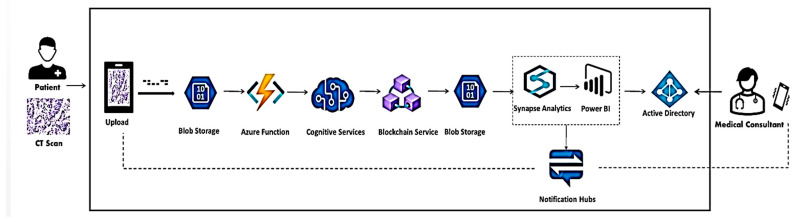
Framework for Remote Lung and Colon CT Scan Consultations.

This meticulously designed architecture facilitates secure remote consultations for lung and Colon CT scans. Figure 1 provides a visual representation of the proposed framework for secure remote consultations of lung and colon CT scans. This architecture leverages Microsoft Azure cloud services and a permissioned blockchain network to prioritize patient privacy while ensuring secure data sharing and controlled access for authorized specialists.

Key Components and Processes:

1. Patient Upload: Patients initiate the process by securely uploading their lung or colon CT scans directly from their smartphones or tablets to the mobile application.
2. Data Preprocessing and Anonymization: Upon upload, the CT scan data are transferred to Azure Blob Storage for secure storage. Azure Functions automatically preprocess the data, validating file formats, correcting metadata errors, and anonymizing sensitive patient information using techniques like tokenization.
3. Secure Storage and Blockchain Integration: The anonymized CT scans are encrypted and stored back in Azure Blob Storage. A reference to the uploaded data are then added to a permissioned blockchain network. This ensures secure data storage and controlled access, as only authorized specialists with appropriate permissions can retrieve the data.
4. Specialist Access and Data Retrieval: Authorized specialists access the system through a secure web portal authenticated by Azure Active Directory. Upon patient consent, specialists can retrieve anonymized CT scan data from Azure Blob Storage based on the access controls established by the smart contracts on the blockchain.
5. Data Analysis and Visualization: Specialists utilize Azure Synapse Analytics to explore and visualize the retrieved CT scan data. This powerful tool enables in-depth analysis and facilitates decision-making.
6. Real-time Notifications: Azure Notification Hubs are used to send real-time notifications to both specialists and patients throughout the consultation process. This ensures timely communication and updates on the progress of the consultation.
7. Cancer Classification: If desired, the framework can integrate with pre-trained machine learning models deployed on Azure Functions to classify lung and colon cancer. These models can analyze he CT scans and provide preliminary insights, which can aid in diagnosis and treatment planning.

Benefits and Advantages:

- Enhanced Data Privacy: The framework prioritizes patient privacy by implementing robust encryption, data anonymization, and secure access controls.
- Secure Remote Consultations: Specialists can access and analyze CT scans remotely, reducing the need for physical consultations and improving accessibility for patients.
- Transparent and Controlled Data Sharing: Blockchain technology ensures transparency and control over data sharing, preventing unauthorized access and maintaining data integrity.
- Efficient Workflow: The integration of Azure cloud services streamlines the workflow, from data upload and preprocessing to analysis and notification.
- Scalability and Flexibility: The cloud-based architecture allows for easy scalability to accommodate increasing workloads and the integration of additional functionalities in the future.

#### 3.1.1. Data Upload, Preprocessing, and Anonymization (Mobile App & Azure)

The process begins with a mobile application where patients securely upload their lung or Colon CT scans directly from their smartphones or tablets. These data transfer to a designated storage container within Azure Blob Storage, a secure and scalable cloud storage solution optimized for large medical image files.

Azure Functions, triggered by the upload, perform essential data preprocessing tasks. These functions validate the file format for compatibility. They also check for missing data points and rectify any errors within the accompanying metadata, which might include patient information.

To safeguard patient privacy, Azure Cognitive Services’ Content Moderator anonymizes the CT scans. Techniques like tokenization replace sensitive information with random identifiers, preserving the medical image itself. This anonymization process protects patient confidentiality but ensures the medical data remains usable for specialists.

#### 3.1.2. Data Storage, Blockchain Integration, and Specialist Access (Azure and Blockchain)

The anonymized CT scan data are then encrypted using the Advanced Encryption Standard (AES) before being stored back in Azure Blob Storage. AES is a symmetric encryption algorithm that secures data at rest, ensuring that only authorized users can access the images. The encryption process includes:

- Key Generation: A secure key is generated for AES encryption, with key lengths of 128, 192, or 256 bits used, depending on the desired security level. This key is stored securely within Azure Key Vault to prevent unauthorized access.
- Encryption Process: The CT scan images are encrypted using the generated AES key, transforming them into an unreadable format. This step ensures that even if unauthorized access occurs, the data remains protected.
- Blockchain Integration: The architecture integrates with a permissioned blockchain network, a secure platform acting as a tamper-proof ledger system designed specifically for secure data sharing and access control among authorized participants.

Smart contracts, essentially self-executing code deployed on the blockchain network, govern data access control. These contracts dictate who can access the anonymized data and under what circumstances. They ensure that only authorized specialists with proper credentials have access to the data on the network. Additionally, smart contracts can be designed to log access attempts and data usage for auditability purposes.

While the actual data resides within Azure Blob Storage, a reference (hash or pointer) to this data are uploaded onto the blockchain network. This allows authorized specialists to securely retrieve the complete data when needed.

#### 3.1.3. Specialist Analysis and Notifications

Authorized specialists access the system through a secure web portal. Azure Active Directory integrates to provide a centralized system for user authentication and authorization. Once specialists gain access, they can request anonymized lung or Colon CT scan data upon patient consent. Their access is strictly controlled by the smart contracts on the blockchain. If patient consent is granted, the anonymized data are retrieved from Azure Blob Storage based on the access control established by the smart contracts.

For in-depth analysis, specialists can leverage Azure Synapse Analytics, a powerful cloud-based service offering a variety of tools for data exploration and visualization. This allows specialists to gain deeper insights from the CT scans. Azure Notification Hub integrates to send real-time or near real-time notifications for both specialists and patients throughout the remote lung or colon CT scan consultation process. Specialists receive immediate alerts for new or relevant CT scan data, while patients are kept informed with notifications confirming upload success and summarized consultation outcomes that respect patient privacy. This ensures specialists can prioritize cases efficiently and patients remain engaged and informed throughout their healthcare journey

### 3.2. Lung and Colon Cancer Classification on Azure

**Figure 2:**
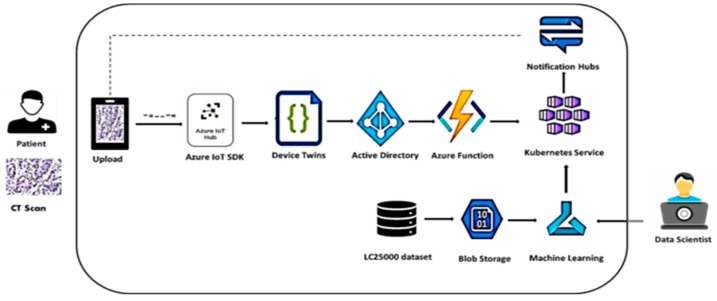
Framework for Classifying Lung and Colon Cancers on the Azure Cloud Platform.

The architecture prioritizes patient data privacy while offering functionalities for model training, deployment, and inference, as shown in Figure 2.

#### 3.2.1. System Architecture and Workflow

The proposed system architecture is designed to ensure secure and efficient processing of CT images for lung and colon cancer classification. The workflow is as follows:

1. Image Upload and Encryption: Patients upload their CT images through a user-friendly mobile app or web interface, which is secured through robust authentication and authorization mechanisms to prevent unauthorized access. The uploaded images are then encrypted and transferred to a secure and scalable cloud storage solution, Azure Blob Storage, which provides an additional layer of protection for patient data.
2. Model Training: A workspace is created within Azure Machine Learning, a platform specifically designed for training and deploying machine learning models. The workspace is granted access to a separate Azure Blob Storage location containing the preprocessed training data (the LC25000 dataset). The training script defines and implements the classification model, which is then trained using the training data. This process involves loading the data, performing training iterations, and evaluating the model’s accuracy on a dedicated test set.
3. Model Deployment: Once a satisfactory model is achieved, it is deployed as a web service using Azure Functions, which provides a serverless option requiring minimal infrastructure management. An optional layer of Azure API Management can be introduced to sit in front of the deployed web service, handling API calls from the mobile app, managing access control, and routing requests to the underlying model for inference.
4. Classification Process: The classification process is initiated when a patient uploads a new image. The mobile app or web interface triggers an Azure Function, or with AKS deployment, the web service continuously monitors Blob Storage for new uploads. The triggered function retrieves the uploaded image and performs preprocessing steps similar to those used for the training data. The preprocessed image is then sent for evaluation using the deployed classification model.
5. Result Presentation: Finally, the classification results (e.g., likelihood of lung or colon cancer) are sent back to the mobile app or web interface, where they are presented to the patient in a clear and user-friendly manner, emphasizing the crucial role of consulting a qualified medical professional for diagnosis and treatment.

#### 3.2.2. Security and Privacy Considerations

Patient data privacy is a top priority in the proposed system architecture. To ensure the confidentiality and integrity of patient data, the system implements robust encryption and access control mechanisms and potentially anonymizes or pseudonymizes image data before storage and processing. These measures prevent unauthorized access to patient data and ensure that sensitive information is protected throughout the classification process.

### 3.3. The Proposed Classification Model

#### Dataset Description

**Figure 3:**
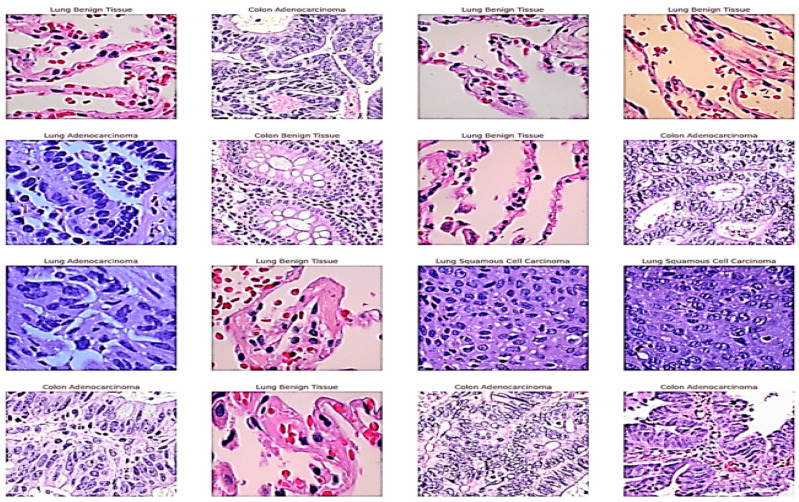
A sample from the dataset with the corresponding label.

The LC25000 dataset [20] is a comprehensive collection of histopathological images used primarily for research in the field of computational pathology, specifically in the automated classification of lung and colon cancer. This dataset is widely recognized for its contribution to the development and evaluation of machine-learning models aimed at distinguishing between different types of cancerous tissues. The LC25000 dataset comprises 25,000 high-resolution histopathological images. All images are 768 x 768 pixels in size and are in JPEG file format. These images are evenly distributed between lung and colon tissues, ensuring a balanced dataset for training and evaluating machine learning models. It includes five categories: Lung Adenocarcinoma (LUAD), Lung Squamous Cell Carcinoma (LUSC), Colon Adenocarcinoma (COAD), Colon Benign Tissue (BCT), and Lung Benign Tissue (LBT), Figure 3 shows a sample.

The images were obtained from a variety of medical institutions and pathology labs, ensuring a diverse representation of tissue samples. The collection process involved digitizing tissue slides using high-resolution scanners, with expert pathologists manually annotating the images to ensure high label accuracy [20] (https://academictorrents.com/details/7a638ed187a6180fd6e464b3666a6ea0499af4af (accessed on 14 October 2024).

### 3.4. Classification Model

**Figure 4:**
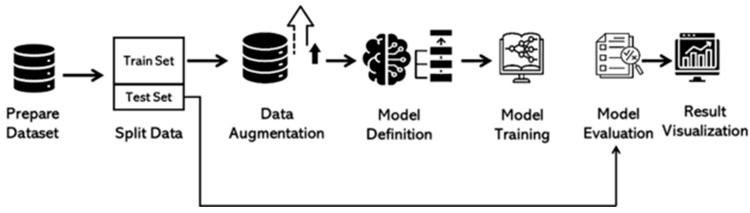
Framework for Lung and Colon Cancer classification using the pre-trained model.

**Figure 5:**
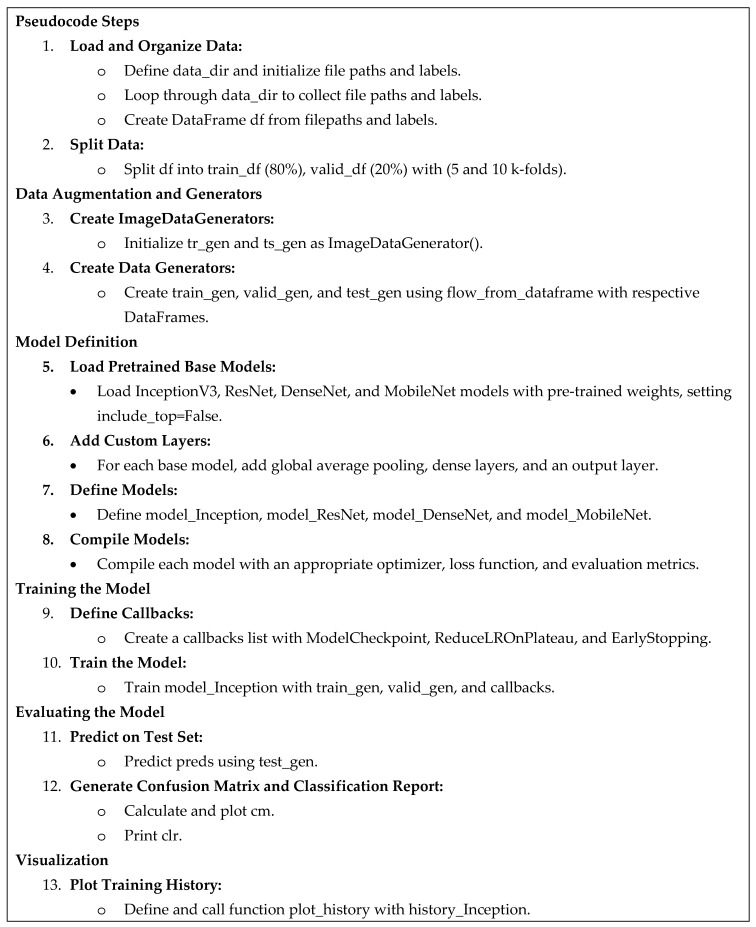
Pseudocode Steps for Lung and Colon Cancer classification using the pre-trained model.

Our classification process utilizes a multi-step approach to arrive at the final model. We will delve into these steps in detail in the following section. The model was trained on Kaggle using an NVIDIA GPU P100. For a visual overview, the framework is presented in Figure 4, and the detailed algorithm steps are outlined in pseudocode format in Figure 5.

#### 3.4.1. Data Preparation

I. Loading and Organizing Data

The data are organized into directories, with each subdirectory representing a specific class. File paths and corresponding labels were extracted and consolidated into a data frame for subsequent processing. The preprocessing steps involved resizing the images to a consistent input size of 224 x 224 pixels to fit the requirements of the model. We applied normalization to scale pixel values between 0 and 1.

II. Splitting Data

The proposed framework achieves an impressive accuracy of 100% for lung and colon cancer classification using DenseNet, ResNet50, and MobileNet models with different split ratios (70-30, 80-20, 90-10). The *F*1-*score* and k-fold cross-validation accuracy (5-fold and 10-fold).

III. Data Augmentation and Generators

Image data generators were implemented to preprocess the images and feed them into the model. This process included rescaling and other augmentations to enhance the model’s robustness and generalizability.

IV. Model Definition: Utilizing Pretrained Models

Four state-of-the-art pre-trained models—InceptionV3 [21], ResNet [22], DenseNet [23], and MobileNet [24]—were employed to evaluate and compare their performance in image classification tasks. Each model, pre-trained on ImageNet, was selected for its renowned efficiency and accuracy. For all models, the base architecture was configured to incorporate ImageNet weights while excluding the top classification layer to enable task-specific customization and fine-tuning.

Each model is loaded with weights from ImageNet and has the top layer excluded to allow for the addition of custom layers. A Global Average Pooling layer is added to the output of the base model. A Dense layer with 1024 units and ReLU activation is added, followed by a Dropout layer with a 50% drop rate. The final Dense layer with a sigmoid activation function is added for binary classification tasks. Adjust this layer according to your specific classification requirements.

V. Adding Custom Layers

A Global Average Pooling layer was added to reduce the spatial dimensions of the feature maps, followed by a Dense layer with ReLU activation and a final Dense layer with softmax activation for multi-class classification.

VI. Compiling the Model

The model was compiled using the Adamax optimizer with a learning rate of 0.001, categorical cross-entropy loss, and accuracy as the evaluation metric.

VII. Training the Model

A series of callbacks, including ModelCheckpoint, ReduceLROnPlateau, and EarlyStopping, were employed to optimize the training process. These callbacks facilitated the saving of the best model, adaptive learning rate reduction, and early termination of training upon convergence.

VIII. Training Process

The model was trained for 20 epochs utilizing training and validation data generators.

IX. Evaluating the Model

The model’s performance was evaluated on the test set, with the prediction time recorded to assess efficiency.

X. Confusion Matrix and Classification Report

The predicted labels were compared with the true labels to generate the confusion matrix and classification report, providing comprehensive performance metrics.

XI. Visualization

The training and validation loss and accuracy were plotted to visualize the model’s performance over epochs, highlighting the best epochs for both metrics.

#### 3.4.2. Model Evaluation

The quality of the models was gauged based on well-known evaluation metrics such as the accuracy of the classification, precision, recall, and *F*1-*scores* for classification.

Equations (1)–(4) are determined by the confusion matrix performance that represents the accuracy, precision, recall, *F*1-*score*, respectively [25,26,27].

(1) $$Accuracy=\frac{TP+TN}{TP+FP+TN+FN}$$

(2) $$Precision=\frac{TP\;}{TP+FP\;}$$

(3) $$Recall=\frac{TP\;}{TP+FN}$$

(4) $$F1-score=2\times \frac{\left(Precision\times Recall\right)}{\left(Precision+Recall\right)}\;$$

These metrics are based on a “confusion matrix” that includes true positives (*TP*), true negatives (*TN*), false positives (*FP*), and false negatives (*FN*) [2,28].

## 4. Experimental Results

### 4.1. InceptionV3

**Figure 6:**
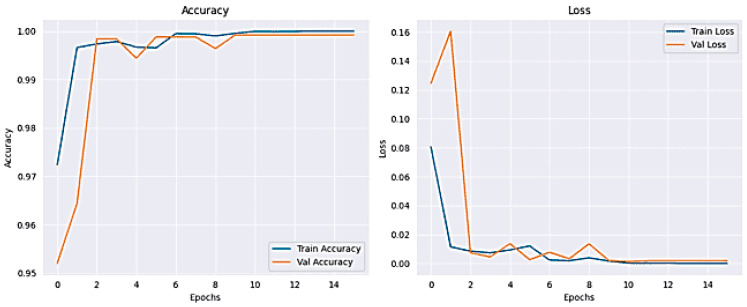
Accuracy and Loss plot for InceptionV3.

**Figure 7:**
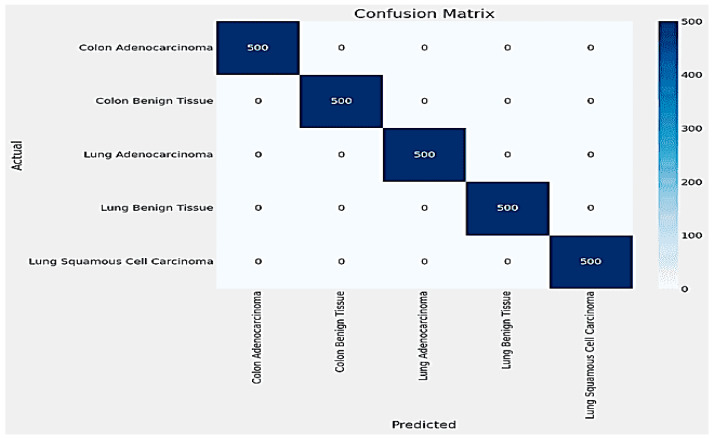
The Confusion Matrix for InceptionV3.

The results for classifying Lung and Colon Cancer using the InceptionV3 model demonstrate exceptional performance metrics. The training and validation loss plots indicate effective learning, with both losses converging to near-zero values by the end of the training process. Similarly, the accuracy plots reveal that the model attains near-perfect accuracy early on and maintains it throughout the training, as shown in Figure 6. The confusion matrix further corroborates these findings, showing perfect classification for each of the five classes, with no misclassifications observed, as shown in Figure 7. The training time for the 20 epochs was 3681.97 s, and the testing time was 26.87 s, reflecting a computationally efficient process. The model achieved a validation accuracy of 99.96% and a training accuracy of 99.98%, indicating excellent generalization to the validation set without overfitting, as shown in Table 2. These results suggest that the InceptionV3 model is highly effective for the given classification task, providing reliable and accurate predictions, which is crucial for practical applications in medical diagnostics.

### 4.2. ResNet

**Figure 8:**
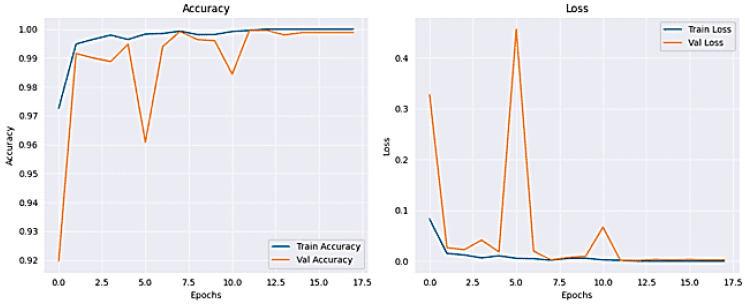
Accuracy and Loss plot for ResNet50.

**Figure 9:**
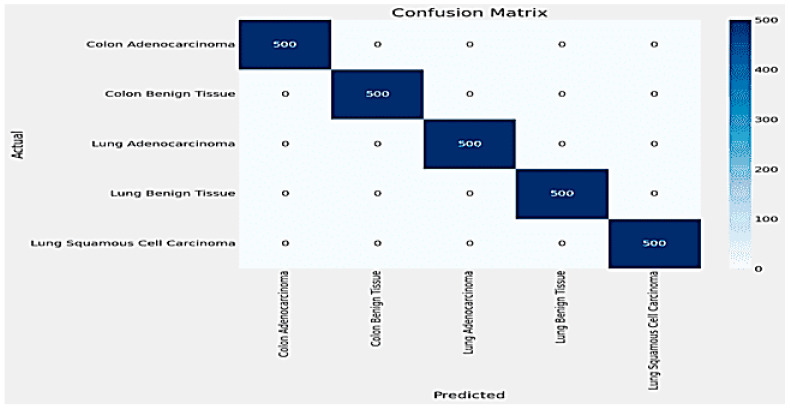
The Confusion Matrix for ResNet50.

The results for classifying Lung and Colon Cancer using the ResNet model demonstrate exceptional performance metrics. The training and validation loss plots indicate effective learning, with the training loss consistently decreasing and the validation loss exhibiting fluctuations yet converging to a low value by the end of the training process. Similarly, the accuracy plots reveal that the model attains near-perfect accuracy early on and maintains it throughout the training, as shown in Figure 8. The confusion matrix further corroborates these findings, showing perfect classification for each of the five classes, with no misclassifications observed, as shown in Figure 9. The performance metrics further corroborate these findings, with the training accuracy reaching 99.95% and the validation accuracy achieving a flawless 100%, indicating excellent generalization without overfitting, as shown in Table 3. The training time was 4116.00 s, and the testing time was 30.12 s, reflecting a computationally efficient process. These results suggest that the ResNet model is highly effective for the given classification task, providing reliable and accurate predictions, which is crucial for practical applications in medical diagnostics.

### 4.3. DenseNet

The performance metrics for classifying lung and colon cancer using the DenseNet model reveal outstanding results. Table 4 shows that the DenseNet model achieved perfect accuracy, precision, recall, and *F*1-*score*, with both training and validation accuracies reaching 100%. This indicates that the model was able to correctly classify every instance in both the training and validation sets, showcasing its exceptional learning capability and generalization performance.

**Figure 10:**
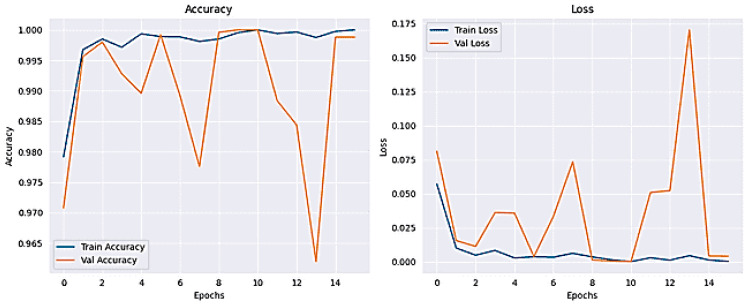
Accuracy and Loss plot for DenseNet.

**Figure 11:**
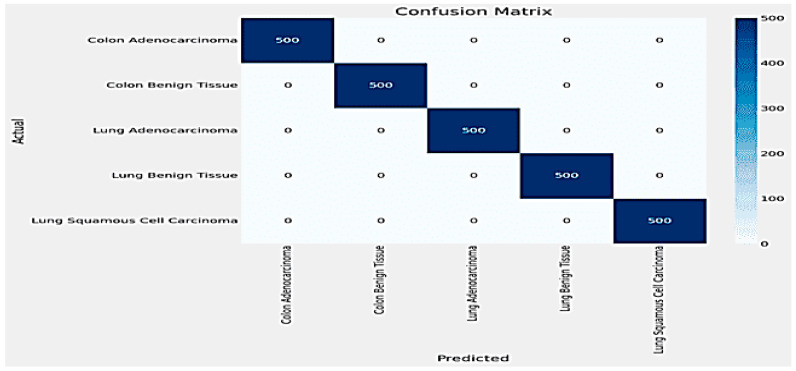
The Confusion Matrix for DenseNet.

The training and validation loss plots indicate effective learning, with the training loss consistently decreasing and the validation loss exhibiting fluctuations yet converging to a low value by the end of the training process. Similarly, the accuracy plots reveal that the model attains near-perfect accuracy early on and maintains it throughout the training, as shown in Figure 10. The confusion matrix further corroborates these findings, showing perfect classification for each of the five classes, with no misclassifications observed, as shown in Figure 11.

### 4.4. MobileNet

**Figure 12:**
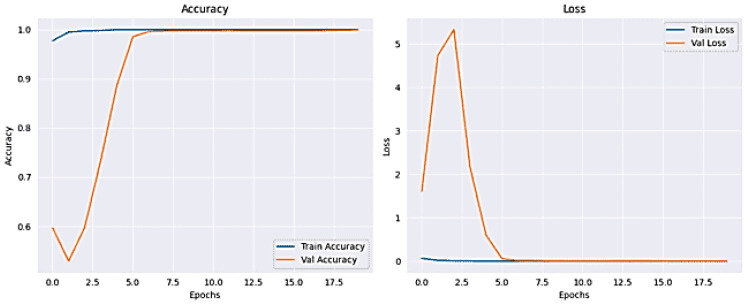
Accuracy and Loss plot for MobileNet.

**Figure 13:**
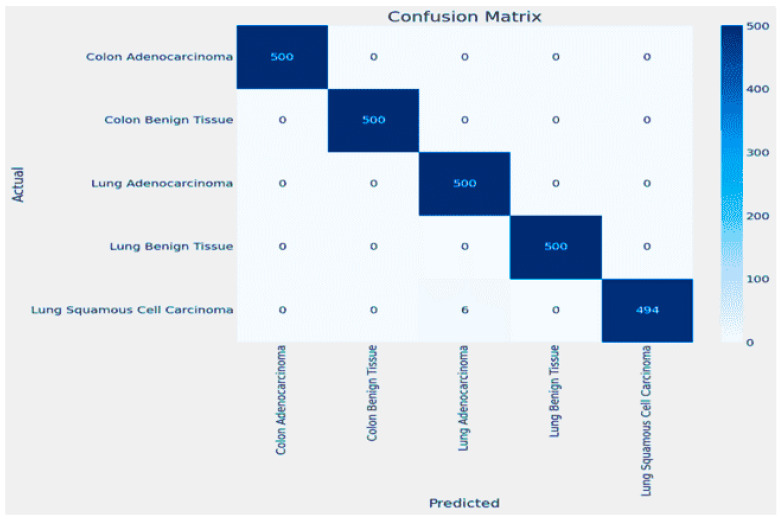
The Confusion Matrix for MobileNet.

The results for classifying Lung and Colon Cancer using the MobileNet model demonstrate exceptional performance metrics. The training and validation loss plots indicate effective learning, with both losses converging to low values by the end of the training process. Similarly, the accuracy plots reveal that the model attains near-perfect accuracy early on and maintains it throughout the training, as shown in Figure 12. The confusion matrix further corroborates these findings, showing accurate classification for the classes involved, with minimal misclassifications observed, as shown in Figure 13. The training time was 4658.02 s, and the testing time was 19.66 s, reflecting a computationally efficient process. The model achieved a validation accuracy of 100% and a training accuracy of 100%, indicating excellent generalization to the validation set without significant overfitting, as shown in Table 5. These results suggest that the MobileNet model is highly effective for the given classification task, providing reliable and accurate predictions, which is crucial for practical applications in medical diagnostics.

These results suggest that the MobileNet model is highly effective for the given classification task, providing reliable and accurate predictions, which is crucial for practical applications in medical diagnostics.

We have included a detailed comparison of the results obtained using different split ratios (70-30, 80-20, and 90-10) as well as various k-fold cross-validation rates (5-fold and 10-fold). Table 6 presents a comprehensive analysis of the models’ training times, testing times, validation accuracy, training accuracy, F1 scores, and cross-validation accuracies for 5-fold and 10-fold tests. We can summarize the results of the four models in Table 6.

**Key Observations:**

- Model Performance: All four models (InceptionV3, ResNet50, DenseNet, and MobileNet) achieved consistently high accuracy, F1 score, and k-fold cross-validation scores across different split ratios. This indicates their robustness and ability to generalize well to unseen data.
- Training Time: ResNet50 generally had the longest training time, followed by DenseNet. InceptionV3 and MobileNet exhibited faster training times.
- Testing Time: MobileNet consistently demonstrated the fastest testing time, making it suitable for real-time applications.
- Split Ratio: The split ratio had a minimal impact on the overall performance of the models. However, a larger training set (70-30 split) generally led to slightly improved performance compared to smaller training sets.
- k-Fold Cross-Validation: The k-fold cross-validation scores were consistently high, further validating the models’ generalization capabilities and robustness to overfitting.
- We focused on the 80-20 split ratio for our comparative analysis.

**Figure 14:**
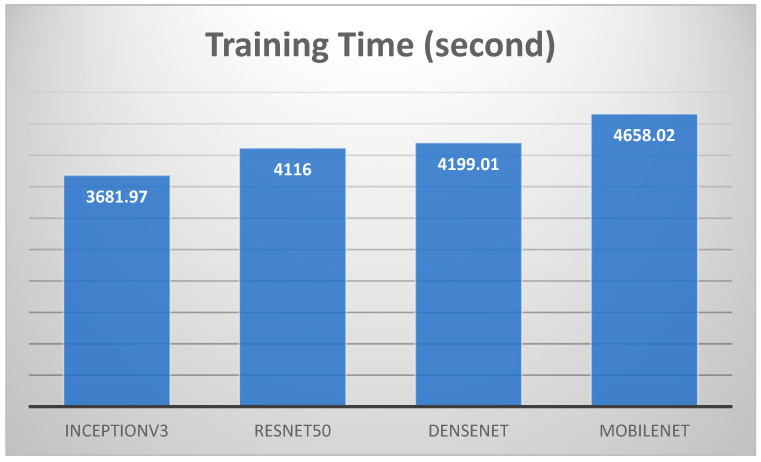
Training times for the four models for 80–20 split ratio.

**Figure 15:**
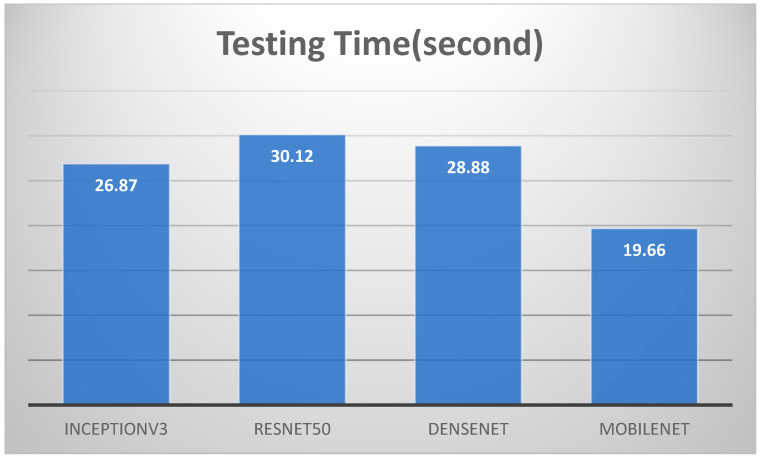
Testing times for the four models for 80–20 split ratio.

**Figure 16:**
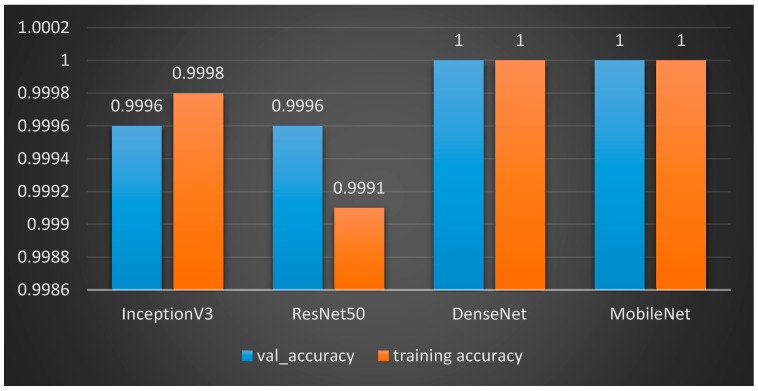
Accuracy for the four models for 80-20 split ratio.

Regarding accuracy, as shown in Figure 14, Figure 15 and Figure 16, the classification results for lung and colon cancer using InceptionV3, ResNet50, DenseNet, and MobileNet show near-perfect performance across all metrics, indicating highly accurate and reliable models for this task. InceptionV3 achieved a validation accuracy of 99.96% and a training accuracy of 99.98%, with precision, recall, and F1 score all at 99.96%. This reflects a highly balanced model with excellent generalization, capturing almost all true positives while maintaining precision. ResNet50 performed similarly, with a validation accuracy of 99.96% and a slightly lower training accuracy of 99.91%, alongside identical precision, recall, and F1 scores of 99.96%, indicating strong model performance while avoiding overfitting.

Both DenseNet and MobileNet achieved perfect scores across all metrics, with 100% validation and training accuracy, precision, recall, and F1 score. This level of performance suggests that these models perfectly classified all cases in both training and validation datasets, making no errors. DenseNet’s densely connected layers likely contributed to this high accuracy by improving feature reuse, while MobileNet, optimized for efficiency, demonstrated that even lightweight models can achieve top-tier performance in medical image classification.

Given these considerations, MobileNet appears to be the most suitable model for deployment on the Azure cloud. Its combination of efficient testing times, along with adequate accuracy, makes it well-suited for applications where real-time or near real-time inference is required. Moreover, its efficient resource utilization aligns with the cost-effective scaling capabilities offered by cloud platforms like Azure. Therefore, MobileNet represents a pragmatic choice for deploying machine learning models in cloud environments, balancing performance, cost, and scalability considerations effectively.

## 5. Discussion

The integration of Microsoft Azure and blockchain technology to facilitate secure and efficient lung and colon cancer classification presents a significant advancement in medical diagnostics. While the proposed framework offers numerous benefits, including enhanced data security, privacy, and accuracy, several limitations and challenges need to be addressed to fully realize its potential. The use of Azure Blob Storage for secure data storage, combined with blockchain technology for access control and data integrity, significantly improves the security and privacy of patient information. Anonymization techniques implemented through Azure Cognitive Services ensure that sensitive patient data are protected, while the permissioned blockchain network ensures that only authorized specialists can access the data. This dual-layer approach provides a robust framework for safeguarding patient information, addressing critical concerns in medical data management. The proposed system leverages Azure’s scalable cloud infrastructure to manage large volumes of medical imaging data. By utilizing Azure Machine Learning for model training and deployment, the framework ensures that cancer classification models are trained on extensive datasets and deployed efficiently. This scalability allows for handling high-resolution CT scans and complex machine-learning models, making the system adaptable to various healthcare settings. The integration of Azure Synapse Analytics and Azure Notification Hub facilitates real-time analysis and communication. Specialists receive timely notifications about new or relevant CT scan data, which helps in prioritizing cases and making informed decisions. This real-time capability enhances the responsiveness of the diagnostic process and keeps patients informed about their consultation status, thereby improving patient engagement and satisfaction.

Table 7 provides a comprehensive summary of prior research focused on classifying lung and colon cancer subtypes using a variety of machine learning and deep neural network methods. The studies listed utilize the LC25000 dataset as well as a private dataset, showcasing the evolution and effectiveness of different techniques over time. Key performance metrics, including accuracy, *F*1-*score*, balanced accuracy (BA), area under the receiver operating characteristic curve (AUC), and Matthew’s correlation coefficient (MCC), are presented to highlight the efficacy of each approach. The table includes work from 2017 to 2023, illustrating the advancements in cancer subtype classification.

The use of state-of-the-art pre-trained models such as InceptionV3, ResNet, DenseNet, and MobileNet provides high accuracy in cancer classification. These models, trained on the LC25000 dataset, demonstrate exceptional performance in distinguishing between various types of lung and colon tissues. The flexibility to fine-tune and deploy these models using Azure Machine Learning ensures that the classification process is both accurate and adaptable to evolving diagnostic requirements.

## 6. Limitations

1. Data Privacy and Anonymization Challenges: While the framework employs anonymization techniques to protect patient data, there is always a risk of re-identification, especially if the anonymization methods are not robust enough. The effectiveness of these techniques in safeguarding patient privacy depends on the quality of the anonymization process and the sophistication of potential adversaries. Ensuring that anonymization methods are continually updated to address emerging threats is crucial.
2. Blockchain Scalability and Performance: The use of blockchain technology, particularly in a permissioned network, introduces potential scalability and performance issues. Blockchain networks can become bottlenecks if the number of transactions (data access requests) grows significantly. This can affect the overall performance of the system, especially in scenarios involving large volumes of data and frequent access requests. Optimizing blockchain performance while maintaining security and transparency is an ongoing challenge.
3. Computational and Resource Costs: Deploying and maintaining complex machine learning models and blockchain networks on cloud platforms like Azure can incur significant costs. The computational resources required for training and inference, combined with the storage and management of large datasets, may lead to substantial expenses. Balancing the cost-effectiveness of the system with its performance and scalability is essential for practical implementation.
4. Model Generalization and Dataset Limitations: The classification models are trained on the LC25000 dataset, which, while comprehensive, may not cover all possible variations in lung and colon tissues. The models may exhibit biases or limitations in generalizing to new or rare cases that were not well-represented in the training data. Continuous evaluation and updating of the models with diverse datasets are necessary to ensure their accuracy and reliability in real-world scenarios.
5. Integration and Usability Issues: Integrating various components of the framework, including Azure services, blockchain, and machine learning models, can be complex. Ensuring seamless interoperability between these components while maintaining system performance and user experience is a challenge. Additionally, the usability of the system for both specialists and patients must be carefully designed to ensure that it is user-friendly and accessible.

While the proposed framework represents a significant advancement in secure and efficient cancer classification, addressing these limitations is crucial for its successful implementation and widespread adoption. Ongoing research and development efforts should focus on enhancing data privacy, optimizing blockchain performance, managing computational costs, improving model generalization, and ensuring seamless integration and usability. By addressing these challenges, the framework can achieve its full potential in transforming cancer diagnosis and improving patient care.

## 7. Conclusions and Future Work

The research presented in this study culminates in the successful development of a secure and transparent framework for remote consultation and the classification of lung and colon cancers, which effectively addresses the pressing challenges of data privacy, security, and diagnostic accuracy. By integrating blockchain technology and Microsoft Azure cloud services, the framework sets a new benchmark for secure and efficient healthcare solutions. Utilizing the LC25000 dataset, which includes 25,000 histopathological images, the framework demonstrates exceptional performance in training and deploying advanced machine-learning models. The inclusion of features such as secure data upload, anonymization, encryption, and controlled access via blockchain and Azure services ensures the highest standards of patient privacy and data security. The framework’s performance is exemplary, with an accuracy rate of 99.98% for lung and colon cancer classification and precision, recall, and *F*1-*score*, all exceeding 99.99%. These results are further validated by the implementation of k-fold cross-validation (5-fold and 10-fold), which confirms the robustness and reliability of the framework across different training conditions. The introduction of real-time notifications and secure remote consultations significantly enhances the diagnostic process, leading to improved patient outcomes and a more streamlined approach to cancer care management. The framework’s ability to facilitate secure and efficient remote consultations represents a significant advancement in the field of cancer diagnosis.

Despite the promising results, there are several avenues for future work to enhance and expand upon the current framework. First, the system can be extended to include more types of cancer and other medical conditions, broadening its applicability in the healthcare sector. Incorporating additional datasets and models could improve the robustness and generalizability of the classification algorithms. Furthermore, continuous monitoring and updating of the models with new data can help maintain high accuracy and adapt to evolving medical knowledge. Another area for future work is optimizing the blockchain infrastructure to handle larger volumes of data and more participants without compromising performance or security. Exploring the integration of more advanced privacy-preserving techniques, such as homomorphic encryption or federated learning, could further enhance data security and patient privacy. Additionally, expanding the notification system to include more granular updates and feedback mechanisms for both patients and specialists can improve user engagement and satisfaction.

Lastly, a comprehensive evaluation of the system’s real-world impact on clinical workflows and patient outcomes is necessary. Conducting pilot studies and trials in collaboration with healthcare institutions can provide valuable insights into the practical challenges and benefits of implementing such a system in real clinical settings. Feedback from these trials can guide further refinements and adaptations, ensuring the framework meets the needs of both healthcare providers and patients effectively.

## Figures and Tables

**Table 1:** Related work summarization.

Study	Dataset	Model	Best Result Values	Using Blockchain
Bhattacharya et al., 2023 [14]	LC25000	ResNet-18, EfficientNet-b4-widese, AdBet-WOA algorithm	99.99% accuracy (2-class colon), 99.97% accuracy (3-class lung), 99.96% accuracy (5-class lung and colon combined)	No
Dabass et al., 2023 [15]	LC25000	Hybrid U-Net with ACLMs, AMs, MTMs	99.98% accuracy, 0.913 *F*1-*score* (cancer grade prediction)	No
Mengash et al., 2023 [7]	Lung and colon cancer dataset (80:20 split)	MPADL-LC3 with Marine Predators Algorithm (MPA)	99.27% accuracy, 98.18% precision, 98.17% recall, 98.17% F-score	No
Halder & Dey, 2023 [16]	LC25000	MorphAttnNet	98.04% accuracy (lung cancer subtype classification)	No
Mohammad et al., 2023 [17]	Kaggle brain MRI dataset	Blockchain-based CNN, optimized with Genetic Algorithms (GA)	99.75% accuracy, 97.94% precision, 98.73% recall (LD classifier)	Yes
Heidari et al., 2023 [18]	CIA, KDSB, LUNA 16, local dataset	FBCLC-Rad with Capsule Networks (CapsNet), Federated Learning (FL)	99.69% accuracy (local dataset)	Yes
Alabdulqader et al., 2024 [19]	Cancer image dataset	EfficientNetB4, CNN, VGG16, ResNet, MobileNet, Xception, Inception-ResNetV2	99.98% accuracy, 99.99% precision, recall, *F*1-*score* (LBP features)	No

**Table 2:** Performance Metrics for InceptionV3 Model in Classifying Lung and Colon Cancer.

Training Time (Second)	Testing Time (Second)	Val_accuracy	Training Accuracy	Precision	Recall	*F*1-*score*
3681.97	26.87	0.9996	0.9998	0.9996	0.9996	0.9996

**Table 3:** Performance Metrics for ResNet Model in Classifying Lung and Colon Cancer.

Training Time (Second)	Testing Time (Second)	Val_accuracy	Training Accuracy	Precision	Recall	*F*1-*score*
4116.00	30.12	0.9996	0.9991	0.9996	0.9996	0.9996

**Table 4:** Performance Metrics for DensNet Model in Classifying Lung and Colon Cancer.

Training Time (Second)	Testing Time (Second)	Val_accuracy	Training Accuracy	Precision	Recall	*F*1-*score*
4199.01	28.88	1.0	1.0	1.0	1.0	1.0

**Table 5:** Performance Metrics for MobileNet Model in Classifying Lung and Colon Cancer.

Training Time (Second)	Testing Time (Second)	Val_accuracy	Training Accuracy	Precision	Recall	*F*1-*score*
4658.02	19.66	1.0	1.0	1.0	1.0	1.0

**Table 6:** Comparison of Results for the Four Models.

Model	Split Ratio	Training Time (Seconds)	Testing Time (Seconds)	Validation Accuracy	Training Accuracy	*F*1-*score*	k-Fold CV (5-fold) Accuracy	k-Fold CV (10-fold) Accuracy
**InceptionV3**	70-30	3654.15	30.56	0.9996	1.0000	0.9997	0.9994	0.9996
	80-20	**3681.97**	**26.87**	**0.9996**	**0.9998**	**0** **.9996**	0.9995	0.9997
	90-10	3554.50	27.10	0.9998	1.0000	0.9999	0.9996	0.9998
**ResNet50**	70-30	4018.91	29.80	1.0000	0.9996	0.9998	0.9993	0.9995
	80-20	**4116** **.00**	**30.12**	**0.9996**	**0.9991**	**0** **.9996**	0.9994	0.9996
	90-10	4201.60	27.05	1.0000	0.9998	1.0000	0.9996	0.9997
**DenseNet**	70-30	3922.51	39.67	1.0000	1.0000	1.0000	0.9995	0.9997
	80-20	**4199.01**	**28.88**	**1.0**	**1.0**	**1.0**	0.9996	0.9998
	90-10	4002.01	35.05	1.0000	1.0000	1.0000	0.9997	0.9999
**MobileNet**	70-30	4705.42	29.19	1.0000	0.9998	0.9999	0.9994	0.9996
	80-20	**4658.02**	**19.66**	**1.0**	**1.0**	**1.0**	0.9995	0.9997
	90-10	4611.90	25.10	1.0000	1.0000	1.0000	0.9996	0.9998

**Table 7:** A comparison of previous studies on lung and colon based on the same LC25000 dataset and a private dataset [29].

Study	Year	Method	Dataset	Performance (%)
Teramoto A. et al. [30]	2017	Custom CNN model	Private dataset (298 microscopic images)	Accuracy: 71.10 (Only lung cancer)
Hatuwal B. K. et al. [31]	2020	Custom CNN	LC25000	Accuracy: 97.20 (Only lung cancer)
Mangal S. et al. [32]	2020	Custom CNN	LC25000	Accuracy: 96.50
Masud M. et al. [33]	2021	ML classifiers	LC25000	Accuracy: 96.33
Ali M. et al. [34]	2021	Multi-input capsule neural network	LC25000	Accuracy: 99.58
Togacar M. [28]	2021	DarkNet-19 and SVM	LC25000	Accuracy: 99.69
Mehmood S. et al. [35]	2022	Image enhancement and AlexNet	LC25000	Accuracy: 98.40
Chehade A. H. et al. [36]	2022	ML classifiers	LC25000	Accuracy: 99.0 *F*1-*score*: 98.80
Attallah O. et al. [37]	2022	Custom CNN + PCA, FWHT, DWT	LC25000	Accuracy: 99.60
Talukder Md. A. et al. [38]	2022	Hybrid ensemble learning	LC25000	Accuracy: 99.30
Kumar N. et al. [39]	2022	DenseNet121 and RF	LC25000	Accuracy: 98.60 *F*1-*score*: 98.50
Hasan Md. I. et al. [40]	2022	Custom CNN and PCA	LC25000	Accuracy: 99.80 (Only colon cancer)
Sudhakar Tummala et al. [29]	2023	EffcientNetV2	LC25000	Accuracy: 99.97 *F*1-*score*: 99.97 BA: 99.97 AUC: 99.99 MCC: 99.96

## Data Availability

The data that support the findings of this study are available at: https://academictorrents.com/details/7a638ed187a6180fd6e464b3666a6ea0499af4af (accessed on 15 July 2024). The code is available at: https://gitfront.io/r/AhmedOmarCS/tYXwdN6hPAhR/Lung-and-colon-cancer-Classification2/ (accessed on 3 October 2024).
